# Association of Alzhemier's disease with hepatitis C among patients with bipolar disorder

**DOI:** 10.1371/journal.pone.0179312

**Published:** 2017-06-16

**Authors:** Herng-Ching Lin, Sudha Xirasagar, Hsin-Chien Lee, Chung-Chien Huang, Chao-Hung Chen

**Affiliations:** 1 School of Health Care Administration, Taipei Medical University, Taipei, Taiwan; 2 Sleep Research Center, Taipei Medical University Hospital, Taipei, Taiwan; 3 Department of Health Services Policy and Management, Arnold School of Public Health, University of South Carolina, Columbia, South Carolina, United States of America; 4 Department of Psychiatry, Taipei Medical University-Shuang-Ho Hospital, Taipei, Taiwan; 5 Department of Psychiatry, School of Medicine, College of Medicine, Taipei Medical University, Taipei, Taiwan; 6 Department & Institute of Physiology, National Yang-Ming University, Taipei, Taiwan; 7 Department of Cosmetic Applications and Management, Mackay Junior College of Medicine, Nursing, and Management, Taipei, Taiwan; 8 Department of Thoracic Surgery, MacKay Memorial Hospital, Taipei, Taiwan; 9 Research Center of Sleep Medicine, College of Medicine, Taipei Medical University, Taipei, Taiwan; Centers for Disease Control and Prevention, UNITED STATES

## Abstract

Associations of hepatitis C virus infection with Alzheimer’s disease have not been studied among higher risk, bipolar disorder patients. This population-based case-control study investigated the risks of hepatitis C virus infection among Alzheimer’s disease patients with bipolar disorder in the years preceding their Alzheimer’s disease diagnosis. We used 2000–2013 data from the Longitudinal Health Insurance Database in Taiwan. Among patients with bipolar disorder, 73 were diagnosed with Alzheimer’s disease (cases), who were compared with 365 individuals with bipolar disorder but without Alzheimer’s disease (randomly selected controls matched on sex, age, and index year with cases). Prior claims (before the diagnosis year/index year for controls) were screened for a diagnosis of hepatitis C virus infection. Conditional logistic regression models were used for analysis. We found that 23 (31.51%) and 60 (16.44%) patients with bipolar disease were identified with a hepatitis C diagnosis among those with and without Alzheimer’s disease, respectively. Compared to controls, patients with Alzheimer’s disease showed 2.31-fold (95% confidence interval = 1.28–4.16) increased risk of hepatitis C infections adjusted for demographics and socio-economic status. Findings suggest an association of Alzheimer’s disease with a preceding diagnosis of hepatitis C infection among patients with bipolar disorder. Findings may suggest a need for increased awareness of and appropriate surveillance for Alzheimer’s disease in patients with bipolar disorder diagnosed with hepatitis C infection.

## Introduction

Bipolar disorder (BD) is a common, disabling mental disorder, with lifetime prevalence rates ranging from 0.8% to 2% [1]. In addition to unusual shifts in mood, energy, and activity levels, impaired cognition is increasingly recognized as a central feature of the BD phenotype. Indeed, growing concerns are being articulated that BD may be associated with cognitive deficits even during euthymic periods, especially executive function and attentional processing [2, 3]. In a systematic review and meta-analysis, Diniz et al. concluded that there is a significantly higher risk of dementia among older adults with a history of BD [4].

Other studies point to an increased risk of infectious diseases among patients with BD likely due to higher-risk behaviors, such as using injectable drugs, having multiple sexual partners and high-risk partners, and infrequent condom use, in addition to the overarching issue of poverty that often accompanies severe mental illness [5–7]. Among the infectious diseases, hepatitis C virus (HCV) infection is a contemporary worldwide public health problem with a high disease burden. According to the World Health Organization, approximately 130–150 million people globally are chronic carriers of HCV, contributing to more than 350,000 deaths annually from hepatitis C-related liver disease [8]. Among patients with BD, a remarkably elevated risk of HCV infection is reported, with estimated infection rates of 10% to 23.3% [9, 10] compared to 1.8% in the general population [11].

HCV infection has serious consequences. In addition to liver-related sequelae (e.g., cirrhosis, hepatic failure, and hepatocellular carcinoma), chronic HCV infection is linked to increased risks of extra-hepatic morbidity, including metabolic, cardiovascular and neurological morbidities [12–14]. Specifically, cognitive impairment is a frequently observed neurological manifestation of HCV infection, attributed as one contributor to lower quality of life among HCV infected persons. Recent literature documents considerable neurocognitive impairment among a segment of HCV-infected patients with minimal or absent liver disease, with the deficits particularly manifesting in concentration and speed of working memory [15, 16]. Furthermore, specifically, HCV infection is reported to increase the risk of dementia, including Alzhemier's disease (AD), after adjusting for alcohol-related disease, liver cirrhosis, hepatitis encephalopathy, and hepatocellular carcinoma [17].

To summarize, previous literature documents that BD is associated with increased risks of both dementia and HCV infection, presenting a major clinical and therapeutic challenge. Although HCV infection is documented to be associated with elevated risk of AD in the general population [15–17], this association has not been explored among patients with BD who are shown to have higher risks of hepatitis C and AD in separate studies. Addressing this knowledge gap may be important for BD patients’ quality of life because early detection of cognitive decline and its associated factors can help mitigate the severe health consequences of dementia.

We conducted a nationwide, population-based study to investigate the risk of incident AD among HCV infected patients with BD compared to BD patients without HCV infection. We hypothesized that for patients with BD, a diagnosis of HCV infection may be a risk factor for a subsequent diagnosis of AD.

## Methods

### Database

This study used 2000~2013 data from the Taiwan “Longitudinal Health Insurance Database 2005” (LHID2005), which includes claims data for 1,000,000 beneficiaries under the Taiwan National Health Insurance (NHI) program. This longitudinal dataset provides an excellent opportunity to track enrollees’ healthcare utilization from 1995, the year of National Health Insurance implementation. Hundreds of research studies have been published based on claims data from Taiwan’s single payer, NHI program [18].

This study was exempt from full review by the Institutional Review Board of Taipei Medical University (TMU-JIRB 201612019) because the LHID2005 consists of de-identified, publicly available secondary data released for research purposes.

### Study sample

We first identified 9239 patients aged more than 18 years outpatient treated for BD between January 2000 and December 2013, with at least one claim showing a BD diagnosis made by a board-certified psychiatrist (ICD-9-CM code of 296.0X, 296.4X, 296.5X, 296.6X, 296.7X, 296.80 or 296.89). Of the 9239 patients with BD, 73 were identified to have a diagnosis of AD (ICD-9-CM code 331.0). Because of concerns about the diagnostic accuracy documented in administrative datasets, we restricted study cases to those who had received prescriptions for acetylcho-linesterase inhibitors (AChEIs; N06DA02, N06DA03, N06DA04) as per the Anatomical Therapeutic Chemical classification system. These 73 BD patients with AD (age range 58~79 years old) were defined as cases for the purpose of the case-control study. We also assigned the date of the first AD diagnosis among all claims as the index date.

The controls were selected from the remaining BD patients without a diagnosis of AD. We randomly selected 365 control BD patients (five per AD patient), matched with the cases on age (<45, 45~64, 65~74, >74 years), sex and index year (year of corresponding case’s first recorded AD diagnosis). Case-control matching was done using the SAS proc surveyselect program (SAS System for Windows, vers. 8.2, SAS Institute, Cary, NC). The controls were selected by matching them to a given case simply on their utilization of any medical service in the same index year as the case. For controls, we assigned their first utilization of medical services in the index year as the index date.

### Exposure assessment

This study explored exposure to hepatitis C infection as the risk factor for subsequent AD among bipolar disease patients. Hepatitis C exposure was identified by the presence of the relevant diagnosis code prior to the index date (ICD-9-CM code 070.41, 070.44, 070.51, 070.54, 070.0, 070.70, 070.71, or V02.62).

### Statistical analysis

The SAS statistical package (SAS System for Windows, Version 8.2) was used for analyses. We used Chi-square tests to explore differences between BD patients with and without AD on demographic characteristics and comorbid medical conditions, including hypertension, diabetes and hyperlipidemia. Conditional logistic regression analysis (conditioned on sex, age group and index year) was performed to compute the adjusted odds of a prior Hepatitis C diagnosis (AOR, 95% confidence interval (CI)) among AD patients relative to patients without AD. A significance level of *p* <0.05 was used.

## Results

Table 1 presents the distribution of cases and controls by socio-economic characteristics and co-morbidities. Cases and controls were similar on monthly income (*p* = 0.652), geographic region of residence (*p* = 0.350), urbanization level (*p* = 0.399), hypertension (*p* = 0.116), diabetes (*p* = 0.488) and hyperlipidemia (*p* = 0.528).

**Table 1:** Demographic characteristics of bipolar disease (BD) patients with and without Alzhiemer’s Disease (AD) in Taiwan (*n =* 438).

Variable	Patients with AD (*n* = 73)		Controls (*n* = 365)		*p* value
	Total no.	%	Total no.	%	
Age (years)					1.000
45~54	1	1.4	5	1.4	
55~64	14	19.2	70	19.2	
65~74	31	42.5	155	42.5	
≥75	27	37.0	135	37.0	
Sex					1.000
Female	47	64.4	235	64.4	
Male	26	35.6	130	35.6	
Monthly Income					0.652
≤NT$15,840	48	65.8	219	60.0	
NT$15,841~25,000	20	27.4	118	32.3	
≥NT$25,001	5	6.9	28	7.7	
Geographic region					
Northern	28	38.4	182	49.9	0.350
Central	20	27.4	78	21.4	
Southern	21	28.8	88	24.1	
Eastern	4	5.5	17	4.7	
Urbanization level					0.399
1 (most urbanized)	22	30.1	108	29.6	
2	21	28.8	123	33.7	
3	13	17.8	37	10.1	
4	10	13.7	51	14.0	
5 (least urbanized)	7	9.6	46	12.6	
Comorbidities					
Hypertension	38	52.1	226	61.9	0.116
Diabetes	25	34.3	110	30.1	0.488
Hyperlipidemia	17	23.3	98	26.9	0.528

Note: The average exchange rate in 2012 was US$1≈New Taiwan (NT)$30.

Table 2 shows the prevalence of hepatitis C predating the index year among cases and controls. We found that among the total sample 83 out of 438 (18.95%) had been diagnosed with hepatitis C prior to the index date; 23 (31.51%) among cases and 60 (16.44%) among controls, a statistically significant difference (*p<*0.001). Conditional logistic regression analysis (conditioned on sex, age group, and index year), also presented in Table 2, shows that the odds of prior hepatitis C for cases was 2.33 (95% CI: 1.33–4.10) relative to controls.

**Table 2:** Prevalence, conditional regression odds ratios (ORs), and 95% confidence intervals (CIs) for hepatitis C among sampled subjects.

Presence of Hepatitis C	Total (*n* = 438)		Patients with AD (*n* = 73)		Controls (*n* = 365)	
	*n*, %		*n*, %		*n*, %	
Yes	83	18.95	23	31.51	60	16.44
No	355	81.05	50	68.49	305	83.56
Conditional logistic regression OR (95% CI)	—		2.33** (1.33~4.10)		1.00	

Notes: The OR was calculated by a conditional logistic regression stratified by sex, age group, and index year.

**p<0.01

Table 3 shows the adjusted odds of prior hepatitis C adjusted for monthly income, geographic region of residence, urbanization level, hypertension, diabetes and hyperlipidemia. The AOR of prior hepatitis C among cases was 2.31 (95% CI = 1.28–4.16) relative to controls.

**Table 3:** Covariate-adjusted odds of prior HCV infection among patients with Alzheimer’s Disease relative to those without Alzheimer’s Disease among patients with bipolar disorder (*n =* 438).

Variables	Presence of AD	
	Adjusted OR	95% CI
Prior presence of Hepatitis C	2.31**	1.28–4.16
Monthly income		
≤NT$15,840 (reference group)	1.00	
NT$15,841–25,000	0.75	0.40–1.41
≥NT$25,001	0.90	0.31–2.66
Urbanization level		
1 (reference group)	1.00	
2	0.75	0.38–1.50
3	1.40	0.60–3.26
4	0.72	0.27–1.92
5	0.51	0.18–1.45
Geographic region		
Northern (reference group)	1.00	
Central	1.89	0.92–3.88
Southern	1.59	0.82–3.08
Eastern	1.73	0.48–6.23
Comorbidities		
Hypertension	0.61	0.35–1.05
Diabetes	1.28	0.70–2.34
Hyperlipidemia	0.81	0.42–1.58

Note

**p<0.01

## Discussion

This is the first epidemiological study in the medical literature to assess the association between prior HCV infection and AD within a population sub-group at high risk for both conditions, bipolar disease patients. In this matched case-control study, we found that 31.5% and 16.4% of AD patients and patients free from AD, respectively, had prior HCV infection. Compared to BD patients without AD, BD patients with AD had a 2.3-fold increased risk of having prior HCV infections, adjusted for the demographic factors of sex, age group, index year, socio-economic factors of income, urbanization, and geographic location, and comorbidities (hypertension, diabetes, and hyperlipidemia).

Our finding of an epidemiological association link between prior HCV infection and AD among bipolar disease patients is consistent with previous reports of a link between HCV infection and neuro-cognitive impairment in the general population. A significant body of literature has documented such evidence even among HCV-infected patients with minimal or no evidence of liver disease [15, 16, 19]. Because we excluded patients with advanced liver fibrosis or cirrhosis, impaired cognition cannot be attributed to potentially co-existing mild hepatic encephalopathy. In addition, studies have shown that the association between HCV infection and cognitive impairment is independent of symptom severity, history of substance abuse, and other neuropsychiatric manifestations, as well as other indices of liver function (e.g., laboratory values of liver function tests, viral load, and genotype) [19]. On the other hand, continuing decline in cognitive function is its chief clinical hallmark of AD [20], the most common type of dementia. In one population-based cohort study, HCV infection was identified as an independent risk factor for AD, regardless of the presence of other medical conditions [17]. It is plausible that this link may be particularly salient for patients with BD, a high-risk population for both HCV infection [5, 7, 10] and dementia, especially AD [21–26].

This study is the first to identify among the BD population, more than double the odds of prior HCV infections among patients with AD compared to unaffected controls,. The observed strength of association in the bipolar disease population is greater than the documented association among the general population (adjusted hazard ratio of 1.38) [17].

Thus far, there is no literature suggesting the underlying mechanisms that may explain the association between HCV infection and cognitive impairment, especially AD. Yet, such a link remains biologically plausible. HCV infection itself may directly affect the central nervous system (CNS) and cause brain dysfunction. Studies indicate that HCV in the CNS elicits neuroinflammation, with specific cytokines shown to change neuroendocrine and neurochemical pathways that are associated with cognitive function [27]. Patients with HCV infection are also found to have abnormal cerebral glucose metabolism and neurotransmission, another mechanism to explain the functional deficits [28, 29]. HCV is also hypothesized to cross the blood-brain barrier and infect the CNS, or indirectly attack the CNS by infecting macrophages and replicating in brain tissue, leading to cognitive abnormalities [30, 31]. In addition to direct CNS impacts, HCV infection is often accompanied by advanced liver disease, illicit drug injection, and other factors (e.g., treatment-related side effects) that may adversely impact cognitive functioning [31]. Additionally, among patients with BD, the experience of manic and/or hypomanic episodes may have neurobiologic underpinnings that may uniquely add to the risk of dementia. Patients with BD are also documented to have displayed higher rates of medical comorbidity (obesity, diabetes), unhealthy behaviors (poor diet, less exercise, smoking), and substance use [32, 33]. These factors may contribute to lower brain and cognitive reserves that typically help coping with the onslaught of dementia, which may translate into elevated risk of clinical dementia. Finally, intrinsic factors associated with BD, particularly genetic susceptibility, may render patients more vulnerable to progressive brain changes and cognitive deficits, exacerbating the link between HCV infection and AD [34].

The magnitude of increased risk of HCV infection among patients with BD is noteworthy. In our study, 16.4% of controls (BD patients without AD diagnosis) had a prior diagnosis of HCV infection, while the HCV infection rate in the general population is estimated as 1.8% [11]. The rate is consistent with previous studies showing that psychiatric populations have a higher risk of acquiring HCV in both developing and developed countries, with prevalence rates in a systematic review of the literature ranging from 0.4% to 38% [35]. The prevalence of HCV infection specifically among patients with BD was far in excess of the general population, 5 to 13-fold higher than the general population [5, 9–11, 36, 37]. The higher HCV risk may reflect the effect of higher likelihood of poverty, risky social environment, high-risk behaviors, and overall poor health and medical care among patients with BD [5–7]. Physicians and care-givers of BD patients should be alert to the unusually high risk for sever liver diseases and associated comorbidities among patients with BD, due to HCV infection.

Our study also highlights the importance of appropriate surveillance for, and early recognition of AD among the elevated-risk population of BD combined with HCV infection. AD is characterized by progressive cognitive impairment that impedes independent functioning, and ultimately requires long-term care. Recognizing AD symptoms early is thus critical to enable administration of medications to manage symptoms and delay deterioration, which is most effective in the early stages of AD. Our study finding of considerably high risk of AD among BD patients who were also chronic carriers of HCV supports alerting psychiatrists and gastroenterologists and family care-givers to identify early AD symptoms among these patients. Furthermore, despite the higher risk of patients with BD for HCV infection, there is a lack of awareness about HCV among providers due to fragmentation of care and the disadvantages faced by patients with severe mental illness [5]. Because HCV treatment has markedly improved over the past decade [38], measures such as screening and testing for HCV antibodies, risk reduction, and referral to medical treatment of HCV infection among patients with BD should become routine practice to optimize their health outcomes.

Our study makes a new contribution by identifying a higher risk of AD among BD patients with prior HCV infection. Being a population-based epidemiological study, selection bias and non-response bias are minimized. The availability of longitudinal data to track prior medical history and use of medical services by the sample patients is an additional strength of the study.

Despite these strengths, the current findings should be interpreted in light of certain limitations. First, the NHI database includes only patients who sought treatment for and got diagnosed with BD, AD, and HCV infection. Individuals with serious mental illness such as BD may experience significant barriers to receipt of non-psychiatric medical care. They are less likely than persons without mental disorders to use various general medical services [39–41]. The majority of patients with severe mental illness may be unware of their HCV infection despite developing cirrhotic liver disease or asymptomatic hepatocellular carcinoma [5]. Therefore prior HCV infection in our study may thus be underdiagnosed, with the potential for differential rates of under-diagnosis among patients with and without AD. Non-differential misclassification of exposures would however, bias our results towards the null. Second, diagnosing AD is challenging due to the lack of objective biomarkers, and a confirmed diagnosis can only be made at autopsy. In our study, a diagnosis of AD was identified from the administrative database through ICD-9-CM codes reported by a psychiatrist. Finally, the claims database lacks data on alcohol consumption, smoking, family history, and laboratory test findings, which may confound our findings. It is reported that the HCV genotypes 1b and 2a are the most prevalent in Taiwan [42]. There was no information on HCV genotypes available in our study, which impedes identification of the genotype that may be linked to AD incidence. However, cognitive impairment is thought to affect persons with both HCV genotypes [43].

The study strengthens the case for future studies to uncover the underlying pathophysiological mechanisms, as well as the role of BD. Studies on the effects of appropriately treating HCV infection among BD patients on AD development and on cognitive may enable strategies for prevention and intervention programs among high-risk psychiatric populations.
